# First Complete Genome Sequence of *Cucumber green mottle mosaic virus* Isolated from Australia

**DOI:** 10.1128/genomeA.00036-17

**Published:** 2017-03-23

**Authors:** Monica A. Kehoe, Roger A. C. Jones, Brenda A. Coutts

**Affiliations:** a Department of Agriculture and Food Western Australia, Crop Protection Branch, South Perth, Western Australia, Australia; b Institute of Agriculture, Faculty of Science, University of Western Australia, Crawley, Western Australia, Australia

## Abstract

We present here the first complete genome sequence of the tobamovirus *Cucumber green mottle mosaic virus* (CGMMV) from Australia, obtained from an infected cucumber plant. Compared with other CGMMV genomes, its closest nucleotide identities were 99.6% to KP772568, 99.3% to KF155229, and 99.1% to DQ767631 from Canada, Israel, and India, respectively.

## GENOME ANNOUNCEMENT

*Cucumber green mottle mosaic virus* (CGMMV) is a tobamovirus in the family Virgaviridae. It has a single-stranded positive-sense RNA genome (1, 2). It was first detected in Europe and then spread to Asia (3). It recently reached North America (4, 5). In 2014, it was detected for the first time in Australia infecting watermelon (Citrullus lanatus) in Australia’s Northern Territory (6). The main hosts are crop species belonging to the Cucurbitaceae, in which it causes symptoms of leaf mottling and mosaic on foliage, plant dwarfing, flesh discoloration, surface mottling, and distortion of fruit (7). Fruit yield and quality losses can be severe in some cucurbit crop species, including watermelon and cucumber (Cucumis sativus) (8, 9). CGMMV is seed-borne, highly contact transmissible, and survives for years in plant debris and soil. It has no known insect vector, but pollination by bees is suspected to play a role in its plant-to-plant spread (9). CGMMV gets introduced to new cucurbit-growing regions by planting infected seed or seedlings (7).

In July 2016, a leaf sample (CGMMV-WA1) was collected from a continental cucumber plant with leaf mottle and distortion symptoms growing in a commercial plastic tunnel house at Geraldton, Western Australia. Total RNA was extracted using the RNeasy minikit (Qiagen) and reverse transcription was performed using the Improm-II reverse transcription kit (Promega), followed by PCR using the GoTaq G2 green master mix (Promega) and primers for CGMMV-specific identification (10). CGMMV was detected in this sample, and a total RNA extract was sent to the Australian Genome Research Facility (AGRF) for library preparation and 150-bp paired-end sequencing on an Illumina MiSeq. In total, 25,224,386 reads were obtained, which were trimmed and *de novo* assembled using CLC Genomics Workbench 8.0.2 (CLC bio). Mapping, alignment, and annotation were performed using Geneious 9.1.4 (Biomatters), as detailed previously (11). The CGMMV genome length obtained was 6,423 nucleotides (nt) with average coverage of 184,764 times. When subjected to a BLAST analysis (12), the closest genomic nucleotide identities were 99.56% to KP772568, 99.3% to KF155229, and 99.1% to DQ767631 from Canada, Israel, and India, respectively. This result for a whole-genome sequence is comparable to the sequence results obtained by Tesoriero et al. (6) for their 1,200-nt-length Northern Territory nucleotide sequence, which a revealed a 99% identity to sequences from Canada (KP772568) and India (DQ767631). Sequencing of other Australian isolates from different cucurbit-growing regions and cucurbit crops would be required to determine the full genetic diversity of CGMMV within Australia.

### Accession number(s).

The complete genome sequence of CGMMV-WA1 has been deposited in Genbank under accession number KY115174.
